# The Influence of Increasing Roughage Content in the Diet on the Growth Performance and Intestinal Flora of Jinwu and Duroc × Landrace × Yorkshire Pigs

**DOI:** 10.3390/ani14131913

**Published:** 2024-06-28

**Authors:** Gaili Xu, Jing Huang, Wenduo Chen, Ayong Zhao, Jianzhi Pan, Fuxian Yu

**Affiliations:** 1 College of Animal Science and Technology & College of Veterinary Medicine, Zhejiang A&F University, 666 Wu Su Street, Hangzhou 311300, China; 13835441083@163.com (G.X.); 19550178717@163.com (W.C.); zay503@zafu.edu.cn (A.Z.); 2 Institute of Virology and Biotechnology, Zhejiang Academy of Agricultural Science, Hangzhou 310021, China; j_huang02@126.com

**Keywords:** roughage diet, Jinwu pig, Duroc × Landrace × Yorkshire pig, roughage tolerance, gut microbiota

## Abstract

**Simple Summary:**

High-fiber unconventional feed, an alternative to conventional feed, is gaining attention as a breakthrough in alleviating the conflict between human and livestock food resources. Incorporating an appropriate amount of crude fiber into the diet can enhance the high-fiber tolerance of the pig gut microbiota, thereby improving the cellulose digesting ability, affecting pig growth performance and gut microbiota, and reducing livestock feed costs. However, there are differences in the tolerance and utilization efficiency of roughage feed among the different breeds. This study aimed to evaluate the changes in Jinwu pigs and Duroc pigs after feeding them diets with an increased roughage content. The results showed that while increasing the roughage content in the diet had a certain impact on the growth rate and feed conversion ratio, it could reduce feed costs and modify the gut microbiota structure. This preliminary study sheds light on the tolerance of Jinwu pigs to roughage and its underlying mechanisms, providing a scientific basis for optimizing feed formulations and improving pig growth performance.

**Abstract:**

The Jinwu pig (JW) is a hybrid breed originating from the Chinese indigenous Jinhua pig and Duroc pig, boasting excellent meat quality and fast growth rates. This study aimed to verify the tolerance of JW to roughage, similar to most Chinese indigenous pigs. In this research, two types of feed were provided to JW and Duroc × Landrace × Yorkshire pigs (DLY): a basal diet and a roughage diet (increasing the rice bran and wheat bran content in the basal diet from 23% to 40%) for a 65-day experimental period. The roughage diet showed an increasing trend in the feed conversion ratio (F/G), with a 17.61% increase in feed consumption per unit weight gain for DLY, while the increase for JW was only 4.26%. A 16S rRNA sequencing analysis revealed that the roughage diet increased the relative abundance of beneficial bacteria, such as *Lactobacillus* and *Clostridium*, while reducing the relative abundance of some potential pathogens, thus improving the gut microbiota environment. After being fed with the roughage diet, the abundance of bacterial genera, such as *Treponema*, *Terrisporobacter*, *Coprococcus*, and *Ruminococcaceae*, which aid in the digestion and utilization of dietary fiber, were significantly higher in Jinwu compared to DLY, indicating that these bacterial genera confer Jinwu with a higher tolerance to roughage than DLY.

## 1. Introduction

Feed costs constitute more than 70% of the total production costs in pig farming. In recent years, the continuous increase in the price of imported feed materials has further reduced the profit margins of pig farmers [1]. Therefore, controlling growth in feed costs and improving feed utilization efficiency are crucial issues that must be prioritized by pig farmers. Researchers have been working on various aspects, such as feed ingredient substitution, reducing feed-to-meat ratios, enhancing effective digestion rates to lower rearing costs, and boosting farming efficiency, thereby fostering the sustainable development of pig farming and stabilizing the domestic pork supply and demand. With the development of pig farming and increasing public concern regarding the quality and safety of animal products, improving pig growth, performance, and health has become a significant topic in the field of animal husbandry. Both feed and gut microbiota are major factors affecting pig growth performance and health. Roughage diets are considered a potential feed improvement strategy because they are believed to have a positive impact on the structure of pig gut microbiota. Currently, various unconventional feed ingredients are being used in pig diet formulations, such as cassava leaves, sweet potato vines, water spinach, rice bran, cassava residue, brewery grains, and tofu residue, among other agricultural and food-processing byproducts. These ingredients are inexpensive and readily available [2]. Research on the potential value of dietary fiber in feed is beneficial for conserving feed resources and is of significant importance for improving pig production performance in the future.

Previous studies have shown that dietary fiber is an anti-nutritional factor that reduces the absorption and utilization of nutrients in pig intestines [3]. As research has progressed, nutritionists have discovered that dietary fiber can have both negative and positive effects on the absorption and utilization of nutrients in pig intestines. Adding dietary fiber to the diet can promote intestinal growth and development, maintain stable gut microbial flora, and preserve a balanced gut ecology, which is essential for intestinal health [4]. Some studies have indicated that adding 5% wheat bran or corn bran to the diet is beneficial for pig growth performance, immune function, and gut microbiota [5], while others have suggested that adding 10% wheat bran to the diet has no significant impact on pig growth performance or gut microbiota [6]. Jie et al. [7] have suggested that adding 30% alfalfa to the diet had no effect on growth performance, but it improved carcass traits and meat quality, which may be related to the response of the gut microbiota, as the gut microbiota plays a crucial role in modulating related phenotypes and gene expression in fattening pigs.

In addition to influencing intestinal development in pigs, roughage diets can inhibit the production of harmful gases. Some fiber substances are metabolized in the intestinal environment to produce volatile fatty acids (mainly acetic acid, propionic acid, and butyric acid) as well as carbon dioxide (CO_2_), H_2_, and methane gases, which, when absorbed, contribute to the dietary energy supply [8]. Microbial fermentation products can promote the proliferation of intestinal and epithelial cells by increasing the intestinal length, mass, and villus height. This, in turn, increases the intestinal surface area to enhance absorption capacity [9]. Butyric acid can improve intestinal defense functions, promote the growth and development of small intestinal villi, enhance pig immune capacity, and facilitate pig growth [10]. Inulin supplementation can improve intestinal barrier integrity [11], and different fiber supplements can regulate intestinal barrier function by stimulating the growth of different bacterial species [12]. The diversity of the gut microbiota is a crucial factor in determining the intestinal microbial balance. Certain components of dietary fiber confer health benefits to pig intestines by promoting the proliferation of beneficial intestinal bacteria and preventing opportunistic pathogens from colonizing [13,14,15]. Different fiber sources have varying effects on bacterial proliferation. The fermentation of resistant starch affects the proliferation of *Bifidobacterium* [16], whereas pectin fermentation stimulates the proliferation of *Lactobacillus* [17]. Grains with high levels of insoluble non-starch polysaccharides stimulate the growth of fiber-degrading bacteria, such as *Ruminococcus* and xylanolytic bacteria [18,19]. Gut microbiota can interact with the host to produce metabolites that play crucial roles in the metabolism and immune stability of intestinal and epithelial tissues and the entire organism [20]. The significant role of gut microbiota metabolites in the intestinal and epithelial barrier after feeding a roughage diet has sparked an increased discussion.

Indigenous Chinese pig breeds have a richer composition of gut microbiota than exotic pig breeds, making them better adapted to roughage diets [21]. Through a long-term struggle with local pathogens, they have developed strong disease resistance and can maintain good health in harsh environments by adapting well to coarse feed. For instance, free-range Tibetan pigs exhibit excellent tolerance to roughage feed, with a digestion ability for fibrous materials in feed that is more than 10% higher than that of penned pigs and even higher than that of DLY, showing superior resistance to coarse feed. The relative abundance of microbes related to fiber digestion in the cecum and colon was significantly higher in Tibetan pigs than in DLY [22]. Compared to Large White pigs, Er-HL pigs showed higher fiber tolerance in terms of growth performance and fiber digestion rates [23].

The main purpose of this research is to explore the differences in growth performance and tolerance of JW and DLY to roughage diets, compare the effects of roughage on the intestinal microbial community structure of different breeds, and evaluate the potential of roughage in improving the health and production efficiency of pigs, especially the application of roughage in breeding fattening pigs. Based on the above research purposes, we believe that due to their different genetic backgrounds, JW and DLY may have differences in their intestinal microbial community structure, which in turn affects their adaptability to roughage diets. By optimizing feed formulas and introducing an appropriate amount of roughage, we can improve the growth performance and health status of pigs while reducing production costs.

## 2. Materials and Methods

### 2.1. Experimental Design and Animal Management

JW and DLY were provided by Ningbo Kuangdai Animal Husbandry Co., Ltd. (Ningbo City, Zhejiang Province, China), and the experiments were conducted at their breeding farm. The farm provides a well-ventilated and hygienic feeding environment. Prior to the experiment, the pens were fumigated and disinfected using formaldehyde and potassium permanganate (in a ratio of 2:1) for 5 d, and ventilation was ensured after fumigation. One hundred JW and 100 DLY piglets were randomly selected from the same batch of piglets born on the farm and raised under identical conditions. The experiment started when the pigs were raised to four months old and ended when they reached six months old. A total of 200 experimental pigs were randomly divided into four groups: a JW basic diet group (CJW), 50 pigs; a JW roughage diet group (RJW), 50 pigs; a DLY basic diet group (CDLY), 50 pigs; a DLY roughage diet group (RDLY), 50 pigs. There were equal numbers of males and females in each experimental group. Each group was divided into 5 pens. This study aimed to investigate the effects of a roughage diet on the gut microbiota of JW and DLY. All experimental pigs had ad libitum access to food and water. The pens were cleaned daily to maintain a dry and ventilated environment, and the health status of the pigs was recorded regularly.

### 2.2. Diet and Feed Composition

The feed required for the experimental pigs was processed by Ningbo Sansheng Biotechnology Co., Ltd. (Ningbo City, Zhejiang Province, China), according to the production feed formulation. The control group (CON) was fed a basic diet, while the roughage group (EG) was given a roughage diet, with the content of rice bran and wheat bran increased from 23% to 40%. The experimental diets were formulated according to the NRC (2012) [24] standards for pig nutritional requirements, and the composition and nutritional levels of the diets are presented in Table 1.

### 2.3. Collection and Processing of Fecal Samples

Fecal samples were collected from six-month-old herd pigs. Clean cotton swabs were used to gently stimulate defecation from the anus of the pigs, and fresh pig feces were scooped into 2 mL sterile centrifuge tubes. The samples were labeled, immediately frozen in liquid nitrogen, and stored. Twenty samples were randomly selected from each experimental group for 16S rRNA data analysis.

### 2.4. 16S rRNA Sequencing

The FastDNA^®^ Spin Kit (Omega Bio-tek, Norcross, GA, USA) for Soil DNA extraction kit was used for initial DNA extraction from the collected samples. DNA purity and concentration were assessed using a NanoDrop2000 (Thermo Scientific Inc., Waltham, MA, USA). Primers 341F (5′-CCTAYGGGRBGCASCAG-3′) and 806R (5′-GGACTACNNGGGTATCTAAT-3′) were used to amplify the V3–V4 region of bacterial 16S rRNA genes. The PCR system consisted of 5 μM primers and 10 ng template DNA, with three replicates for each sample. PCR products from the same sample were pooled, purified using the Axy Prep DNA Gel Extraction Kit (Axygen Biosciences, Union City, CA, USA) after electrophoresis on a 2% agarose gel, and quantified using the Quantus™ Fluorometer (Promega, Madison, WI, USA). Library construction was performed using the NEXTFLEX Rapid DNA-Seq Kit (Bioo Scientific, Austin, TX, USA), and sequencing was performed using the Illumina Nova Seq PE250 platform (Illumina, San Diego, CA, USA).

### 2.5. Statistical Analysis

Raw data were entered into Microsoft Excel 2012, and independent *t*-test samples were performed using SPSS 27.0.1. Statistical significance was set at *p* < 0.05. All data analyses were performed using the Mega Biotech Cloud Platform (https://cloud.majorbio.com).

Fast software (version 0.19.6) was used for quality control of the raw sequencing reads, and the FLASH software (version 1.2.11) was used for read merging. The UPARSE software (version 7.1) was used for OTU clustering with 97% similarity. The sequence count of all samples was rarefied to 39,684, and after rarefaction, the average sequence coverage per sample was 99.09%. Taxonomic annotation of the OTUs was performed using the RDP classifier against the Silva 16S rRNA gene database (v138), with a confidence threshold of 70%. The composition of the microbial community in each sample was analyzed at different taxonomic levels.

## 3. Results

### 3.1. Impact of Roughage Diet on the Growth Performance of JW and DLY

After 65 d of the feeding trial, the growth performance of the experimental pigs was measured and calculated. As shown in Table 2 and Table 3, there were no significant differences in the initial body weight between the roughage and control groups of JW and DLY (*p* > 0.05). HDLY showed a trend of an increased feed conversion ratio (F/G) compared to the control group (a 17.61% increase in feed per unit weight gain), whereas HJW showed a lower trend of change than DLY (a 4.26% increase in feed per unit weight gain). Compared with their respective basic diets, the average daily gain (ADG) and average daily feed intake (ADFI) of JW and DLY showed no significant differences (*p* > 0.05). A further two-way ANOVA analysis on the ADG of the four experimental groups was conducted, and the results are shown in Table 4. The model difference was significant, indicating that the two-factor model of breed and diet designed in the study had a highly significant effect on the ADG trait (*p* < 0.01). Among them, the variance attributed to the breed accounted for 91.13%, the variance attributed to the diet accounted for 7.85%, and the variance attributed to the interaction between the breed and diet accounted for 1.02%. Specifically, the effect of the breed on ADG reached a highly significant level (*p* < 0.01), while the effect of the diet on ADG reached a significant level (*p* < 0.05). However, the interaction between the breed and diet did not reach a significant level.

### 3.2. Effect of Roughage Diet on the Gut Microbiota Diversity in JW and DLY

**Figure 1:**
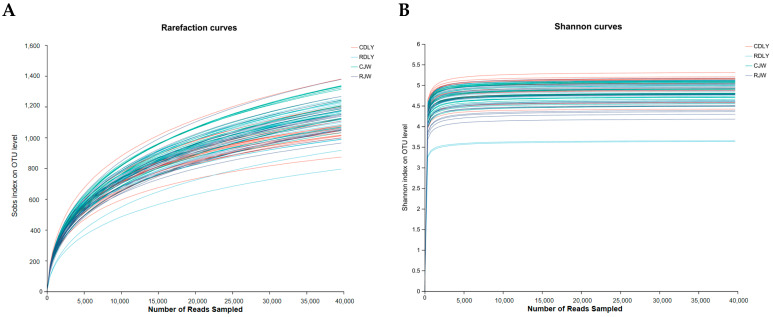
Rarefaction curves. (**A**) The Sobs index curve tends to flatten out, indicating that the sequencing data volume for the four groups is reasonable. (**B**) The Shannon index curve also tends to flatten out, indicating that the sequencing data volume for the four groups is reasonable.

**Figure 2:**
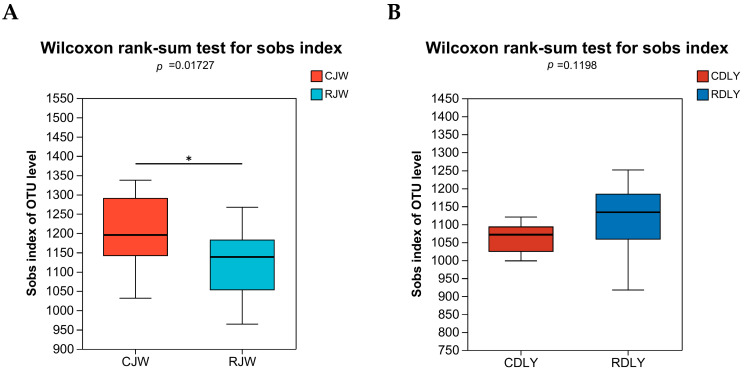
Changes in Sobs index of bacterial communities in pig feces with roughage feeding. (**A**) The species richness within the community of the RJW was significantly lower than that of the CJW (*p* < 0.05). (**B**) There was no significant change in the species richness within the community of the RDLY. *: There is a significant difference between the two groups.

**Figure 3:**
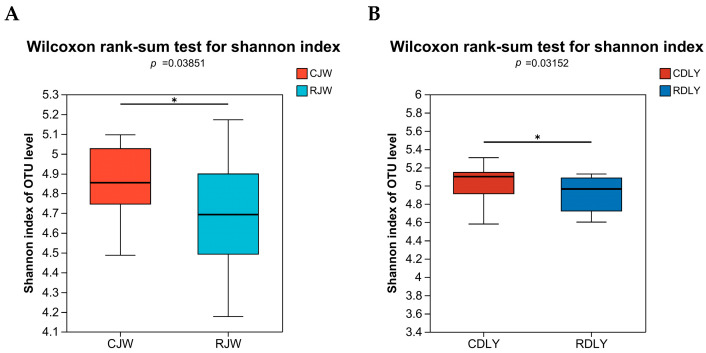
Changes in Shannon index of bacterial communities in pig feces with roughage feeding. (**A**) The species richness and evenness within the community of the RJW are significantly lower than those of the CJW (*p* < 0.05). (**B**) The species richness and evenness within the community of the RDLY are significantly lower than those of the CDLY (*p* < 0.05). *: There is a significant difference between the two groups.

**Figure 4:**
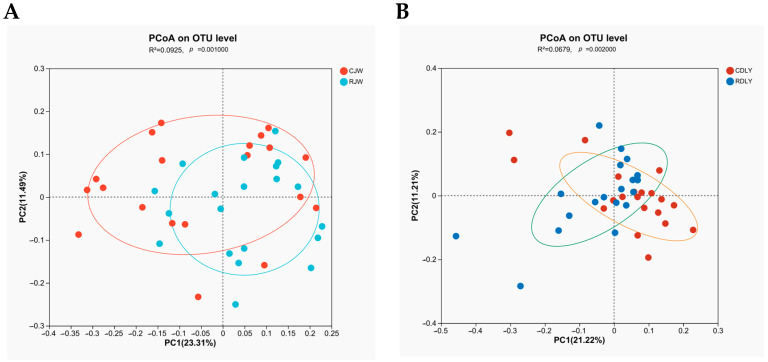
The impact of roughage feeding on the beta diversity of bacterial communities in pig feces. (**A**) There are significant differences in the bacterial communities between the RJW and the CJW (*p* < 0.05). (**B**) There are significant differences in the bacterial communities between the RDLY and the CDLY (*p* < 0.05).

With an increase in sequencing reads, the curves of the Sobs and Shannon indices gradually flattened, indicating that the sequencing depth in this experiment was sufficient to reflect the vast majority of microbial diversity information in the samples and that the amount of sequencing data was reasonable, as depicted in Figure 1A,B. The α diversity of gut microbiota was analyzed using the Sobs and Shannon indices. The Sobs and Shannon indices of RJW showed a significant decrease (*p* < 0.05), as depicted in Figure 2A,B, whereas the Sobs index of RDLY decreased slightly but not significantly (*p* < 0.05), and the Shannon index showed a significant decrease (*p* < 0.05), as depicted in Figure 3A,B. PCoA results indicated that the microbial community structures of RJW and CJW, as well as RDLY and CDLY, differed, as depicted in Figure 4A,B.

### 3.3. Impact of Roughage Diets on the Species Composition of Intestinal Microbiota in JW and DLY

**Figure 5:**
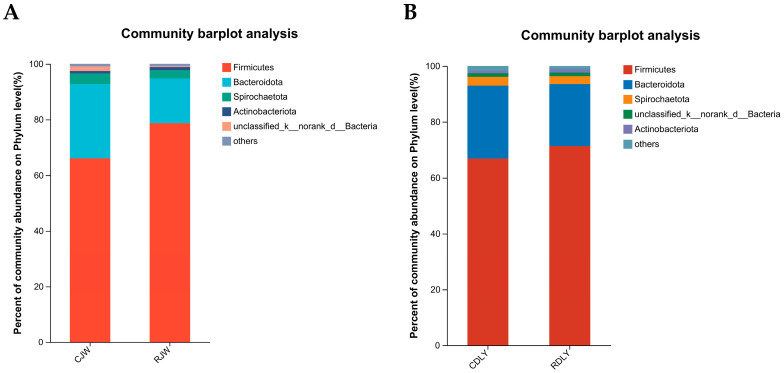
The influence of roughage feeding on the bacterial flora at the phylum level in JW and DLY (**A**) The relative abundance of Firmicutes and Actinobacteria in the RJW significantly increased compared to the CJW (*p* < 0.05). (**B**) The relative abundance of Actinobacteria in the RDLY significantly increased compared to the CDLY (*p* < 0.05).

**Figure 6:**
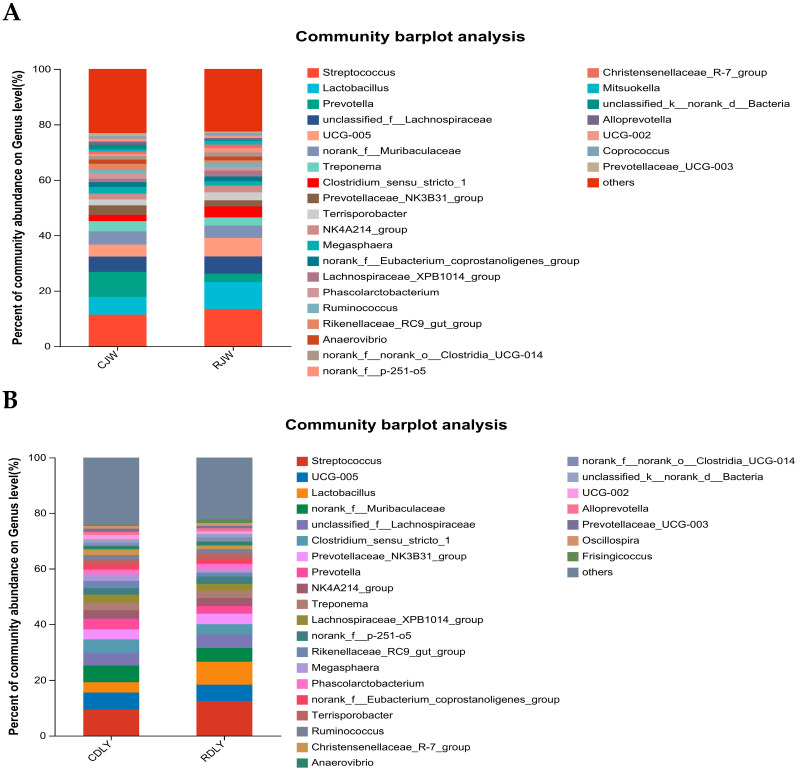
The influence of roughage feeding on the bacterial flora at the genus level in JW and DLY (**A**) The relative abundance of bacterial genera such as *Clostridium_sensu_stricto_1*, *Christensenellaceae_R-7_group*, and *Prevotellaceae_UCG-001* in the RJW significantly increased compared to the CJW (*p* < 0.05). (**B**) The relative abundance of bacterial genera such as *Streptococcus* and *Lactobacillus* in the RDLY significantly increased compared to the CDLY (*p* < 0.05).

The relative abundance and proportion of various taxa at the phylum level can be visually observed from the species annotation results, highlighting the changes in the composition of the intestinal microbiota after feeding roughage diets, as depicted in Figure 5A,B, where abundances of less than 0.01% were merged. At the phylum level, the intestinal microbiota was divided into 17 phyla, with Firmicutes, Bacteroidota, Spirochaetota, and Actinobacteria being the dominant phyla. The changes in the relative abundance and proportion of taxa at the genus level are shown in Figure 6A,B, where abundances of less than 0.01% were merged. A total of 28 genera were identified in the intestinal microbiota at the genus level. The top five dominant genera in CJW at the genus level were *Streptococcus*, *Lactobacillus*, *Prevotella*, *UCG-005*, and *Treponema*; in RJW, the top five dominant genera were *Streptococcus*, *Lactobacillus*, *Prevotella*, *UCG-005*, and *Clostridium_sensu_stricto_1*. In CDLY, the top five dominant genera were *Streptococcus*, *UCG-005*, *Clostridiumsensustricto1*, *Lactobacillus*, and *Prevotella*, while in RDLY, the top five dominant genera were *Streptococcus*, *UCG-005*, *Lactobacillus*, *Clostridiumsensustricto1*, and *PrevotellaceaeNK3B31group*. Compared to the basic diet group, RJW showed an increase in the abundance of *Streptococcus*, *Lactobacillus*, and *UCG-005* genera, whereas the proportion of *Prevotellaceae_NK3B31_group* and *Spirochaetota* genera decreased. RDLY exhibited an increase in the abundance of *Streptococcus*, *Lactobacillus*, and *PrevotellaceaeNK3B31group* genera, whereas the proportions of *UCG-005* and *Clostridiumsensustricto1 genera* decreased.

**Figure 7:**
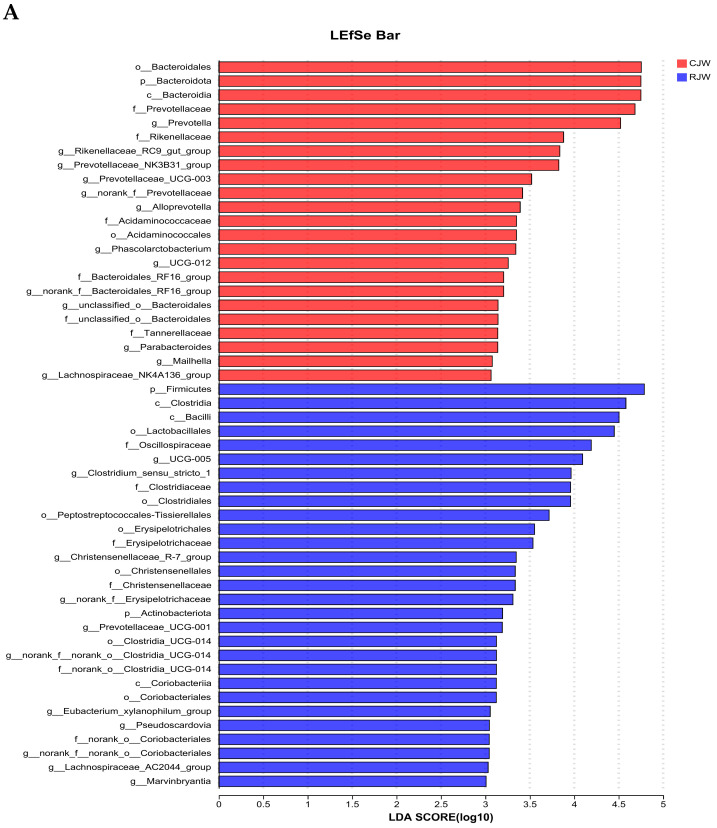

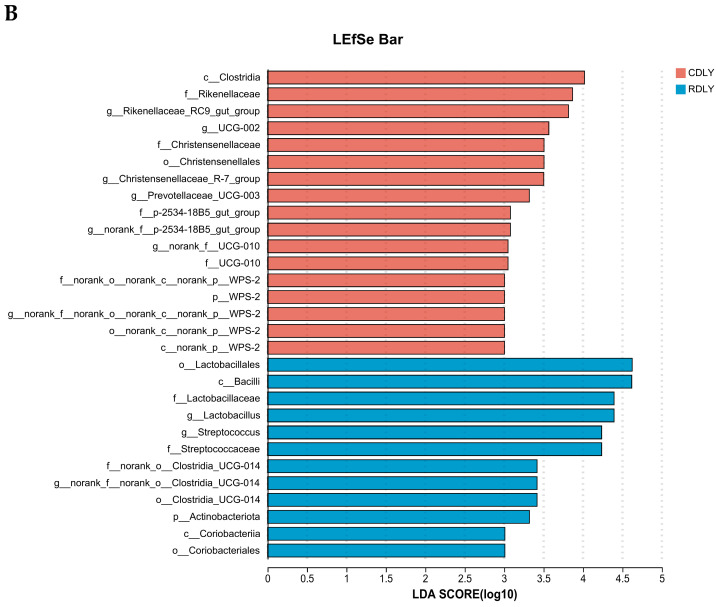

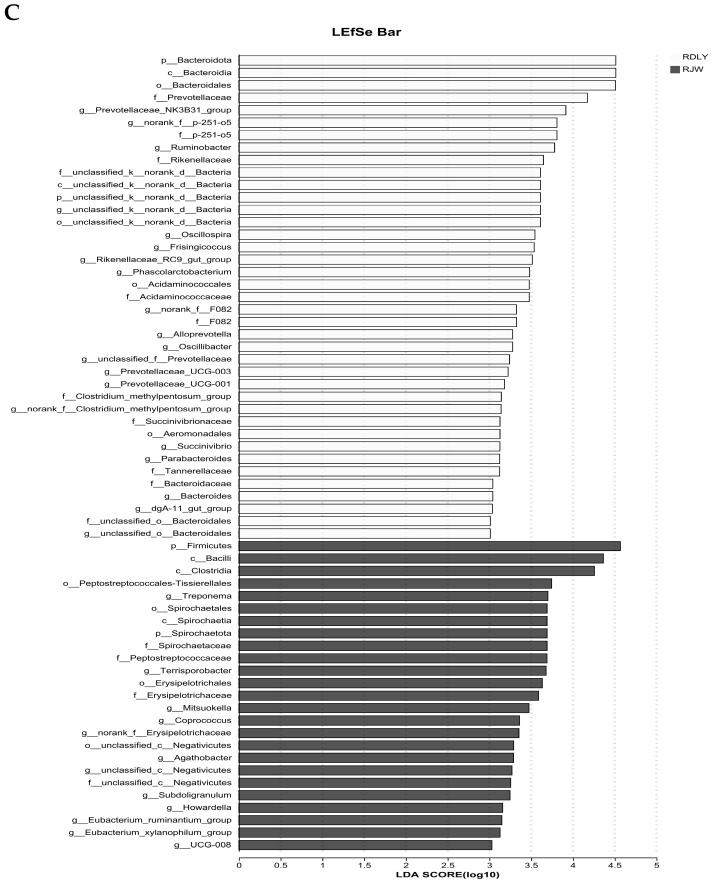
Linear Discriminant Analysis (LDA) Plots for Four Groups (**A**) In the RJW compared to the CJW, the microbiota such as Firmicutes, Clostridia, Lactobacillales, and Clostridiales were significantly enriched. (**B**) In the RDLY compared to the CDLY, the microbiota such as Lactobacillales, Bacilli, and Streptococcus were significantly enriched. (**C**) In RJW compared to RDLY, the microbiota such as *Treponema*, *Terrisporobacter*, *Coprococcus*, and *Ruminococcaceae* were significantly enriched.

A linear discriminant analysis (LDA) was used to analyze differences in microbial communities between groups and to identify species that exhibited significant differences between groups, revealing key communities within each group, as depicted in Figure 7A–C. Bacteroidota, Prevotellaceae, and Rikenellaceae were significantly enriched in CJW, with high LDA scores for fiber-degrading bacteria, such as Bacteroidota and Prevotellaceae, indicating a significant difference in their relative abundance between CJW and RJW. Firmicutes, Clostridia, Bacilli, Lactobacillales, Oscillospiraceae, and Clostridiales were significantly enriched in the RJW. Clostridia, Rikenellaceae, and Prevotellaceae were significantly enriched in the CDLY group. Lactobacillales, Bacilli, and Streptococcus were significantly enriched in RDLY, with substantial differences in their abundance. In RJW, compared to RDLY, the microbiota such as *Treponema*, *Terrisporobacter*, *Coprococcus*, and *Ruminococcaceae* were significantly enriched.

## 4. Discussion

As an important component of feed, it is difficult for pigs to digest and absorb roughage. Despite the low energy utilization rate of roughage, moderate amounts can stimulate intestinal peristalsis and improve digestion and absorption in pigs. In addition, roughage increases the volume of feed, enhances satiety, and improves feed efficiency [25]. Studies have shown conflicting results regarding the effect of increasing roughage content in the diet on the growth performance of livestock and poultry. Some studies suggest that increasing the roughage content of the diet can reduce growth performance, whereas others argue that it has no significant effect on pig growth performance [26]. These contradictory results may stem from differences in the roughage sources, composition, and content [27,28]. For instance, adding 4.3% wheat bran to the diet improved the growth performance of Xiangcun pigs [29], and pigs fed roughage diets showed higher daily weight gain than those fed low-fiber diets [30]. Roughage diets can improve the growth performance of growing and fattening pigs by reducing intestinal oxidative stress and maintaining intestinal health [31].

In this experiment, the intake of roughage by JW and DLY had no significant impact on ADFI (average daily feed intake). However, it led to an increasing trend in the feed conversion ratio (F/G), with the increase in feed consumption per unit weight gain for DLY being 17.61%, while for JW, it only increased by 4.26%. One possible reason for these results is that the pigs in the experiment had ad libitum access to feed. As the roughage content in the diet increased, the digestible energy content decreased, leading to a substantial increase in feed intake to maintain a normal metabolism and internal balance. Therefore, pigs fed roughage diets had a higher feed-to-weight ratio [6]. Another reason could be the differences between pig breeds. Jin Hua pigs are an ancient local breed with a high tolerance for roughage. As the roughage content of the diet increased from 3.86% to 5.92%, the ADG of Jin Hua pigs significantly improved [32]. Since JW has a 37.5% lineage of Jin Hua pigs, it may have inherited their tolerance for roughage, enabling it to better digest and utilize the energy and nutrients from the roughage. This experiment conducted a two-factor analysis on the daily weight gain trait and found that the breed had the greatest impact on daily weight gain. The daily weight gain of DLY was significantly higher than that of JW. The effect of daily feed on daily weight gain was second. Roughage feed had an adverse effect on the daily weight gain of both JW and DLY. The daily weight gain of JW decreased by 6.85%, and the daily weight gain of DLY decreased by 7.61%, indicating that JW has a better tolerance to roughage feed.

The gut microbiota, as a dynamic equilibrium system, is influenced by various factors and participates in multiple physiological cycles to help the host maintain an internal balance at different growth stages [33]. Analysis of the impact of dietary factors on the gut microbiota composition revealed that dietary fiber may be the primary driving factor determining the composition of the gut microbiota [34]. Adding an appropriate amount of roughage to the diet can help control feed costs and promote changes in the tolerance of the gut microbiota to roughage, thereby improving the roughage digestion capacity. In studies on the tolerance of Chinese local pig breeds to roughage, Hanqing et al. [35] found that adding 30% fresh polymerized grass to the diet of pregnant sows significantly improved reproductive performance. In contrast, feeding growing and fattening Sujiang pigs a diet containing 15% fresh polymerized grass did not significantly affect their growth performance.

To explain the roughage tolerance characteristics of local pig breeds from the perspective of gut microbiota and to clarify the characteristics of the JW gut microbiota, we conducted a fecal microbial analysis of different experimental groups. In this study, JW and DLY showed changes in the gut microbiota after consuming large amounts of roughage. The Sobs and Shannon indices of RJW were significantly lower than those of CJW, whereas the Sobs index of RDLY was slightly higher, but not significantly, and the Shannon index was significantly lower than that of CDLY. This may be due to genetic factors; different pig breeds have varying abilities to adapt to roughage diets, resulting in different changes in the gut microbiota species diversity. The relative abundances of Firmicutes and Actinobacteria in RJW significantly increased, whereas the relative abundance of Bacteroidetes decreased. The relative abundances of *Clostridium* and *Lactobacillus* were significantly higher in RJW than in CJW, which is consistent with the results of Pan et al. [36].

In DLY fed the roughage diet, the relative abundance of Actinobacteria increased significantly, whereas that of Desulfobacterota decreased significantly. The abundance of *Lactobacillus*, *Clostridium*, and *Streptococcus* increased significantly. Many studies have indicated that differences in pig breeds can have different effects on the composition of gut microbiota. Indigenous Chinese pig breeds have a more diverse gut microbiota composition than imported Western pig breeds [37]. Bacteria from the phylum Firmicutes can ferment carbohydrates into various short-chain fatty acids (SCFAs) that are beneficial for gut health. They provide energy to the intestinal cells, maintain intestinal mucosal health, and protect the intestinal barrier [38]. In ruminants, the abundance of Firmicutes is typically higher than that of Bacteroidetes [39]. *Clostridium* species primarily utilize indigestible polysaccharides, and most of their metabolites have numerous benefits for gut health [40]. *Lactobacillus*, an important probiotic in the gut, is crucial for maintaining overall health. These probiotics can produce beneficial substances, such as vitamins, aid in the degradation of large protein molecules and lactose, produce small peptides, amino acids, and lactic acid, and promote biological absorption. Moreover, they can inhibit the proliferation of harmful bacteria, maintain the gut microbiota balance, and reduce the occurrence of piglet diarrhea. Fiber- and polyphenol-rich plant foods can also sustainably increase the abundance of *Lactobacillus*, providing antimicrobial and anti-inflammatory effects as well as cardiovascular protection [41]. Bacteria from the *Prevotella* genus can degrade various soluble polysaccharides, such as oligosaccharides, pectin, starch, and xylan. Their metabolic products, including acetate, propionate, and succinate, help lower intestinal pH, maintain the gut microbiota balance, and improve feed efficiency in animals [42]. *Treponema*, which is present in large proportions in the gastrointestinal tract of ruminants, can degrade pectin substances in fibers, is closely related to cellulose-degrading bacteria, and utilizes the carbohydrates released during fiber degradation. This species contributes significantly to lignocellulose degradation in the rumen [43]. *Terrisporobacter*, an acetogenic bacterium, is a fermentative anaerobic bacterium that can ferment substances such as glucose, fructose, sorbitol, and cellulose [44]. It plays an important role in the degradation of organic matter during composting and promotes humification [45]. *Coprococcus* is associated with butyrate production [46], and its abundance increases in animals fed high-resistant starch diets [47]. It belongs to the *Lachnospiraceae* family and is classified under *Clostridium* cluster XIVa, which contributes to immune homeostasis and exhibits anti-inflammatory effects [48]. *Ruminococcaceae* is a dominant bacterial genus in ruminants, containing various genera that utilize different cellulose degradation strategies in the digestive tract to degrade fibers, produce cellulase enzymes to hydrolyze plant fibers, and accumulate SCFAs through fermentation [49]. The abundance of these genera in RJW was significantly higher than that in RDLY, and they were all beneficial for the digestion and utilization of dietary fiber. Therefore, it is speculated that these genera are one of the reasons why JW exhibited better tolerance to roughage than DLY. This may also explain why JW’s feed conversion ratio (F/G) per unit weight gain of JW increased by only 4.26% after feeding a roughage diet, whereas that of DLY increased by 17.61%.

## 5. Conclusions

Feeding a roughage diet did not significantly affect the average daily weight gain and average daily feed intake of JW but resulted in a 17.61% increase in the feed conversion ratio (F/G) per unit weight gain for DLY, while JW only increased by 4.26%, indicating differences between breeds. A roughage diet may alter the interactions between microorganisms and the intestinal environment, leading to changes in the species richness and diversity of the gut microbiota. The roughage diet changed the gut microbiota structure of JW and DLY, increasing the relative abundance of beneficial bacteria, such as *Lactobacillus* and *Clostridium perfringens*, while reducing the relative abundance of some potential pathogens, improving the gut microbiota environment and making it more adaptable to a roughage diet.

Genetic background leads to differences in gut microbiota structure, resulting in different changes in microbial communities after feeding a roughage diet and varying degrees of fiber digestion and utilization. Among these, the abundances of several genera, such as *Treponema*, *Coprococcus*, and *Ruminococcaceae*, were significantly higher in RJW than in RDLY, all of which contributed to the digestion and utilization of fiber in the diet. Therefore, it is inferred that these genera are one of the reasons why JW exhibited better tolerance to roughage than DLY.

## Figures and Tables

**Table 1:** Composition and nutritional content of experimental diets.

Item	Diet Composition	
	CON	EG
Ingredients		
Corn	60.00	48.00
Soybean meal	12.50	8.00
Rice bran	15.00	30.00
Wheat bran	8.00	10.00
Fish meal	0.50	0
Premix ^1^	4.00	4.00
Nutritional Level		
Dry matter (%)	83.08	83.34
Metabolizable energy (MC/kg)	2.89	2.83
Digestible energy (MC/kg)	3.11	3.04
Crude protein (%)	14.79	13.90
Crude fiber (%)	3.27	4.19
Starch (%)	41.92	38.31
Neutral detergent fiber	15.11	19.74
Acid detergent fiber	7.07	9.50
Lignin	2.46	3.27
Calcium	0.11	0.09
Total phosphorus	0.58	0.78
Utilizable phosphorus	0.17	0.17
Lysine	0.66	0.60
Methionine	0.23	0.22
Threonine	0.54	0.50
Tryptophan	0.16	0.15
Isoleucine	0.56	0.54
Leucine	1.26	1.14
Valine	0.70	0.71
Arginine	0.95	0.93
Histidine	0.39	0.38
Phenylalanine	0.72	0.68
Price (RMB/ton)	2666	2165

Abbreviations: CON, basal diet group; EG, roughage group. ^1^ The premix was sourced from Ningbo Mingxing Feed Co., Ltd. (Ningbo City, Zhejiang Province, China). It provides the following vitamin content per kilogram of feed: Vitamin A 60–160 KIU; Vitamin D3 20–60 KIU; Vitamin E 300 IU; Vitamin K3 20–60 mg; Vitamin B1 330 mg; Vitamin B2 80 mg; Vitamin B6 50 mg; Vitamin B12 0.3 mg; Niacin 300 mg; Pantothenic acid 160 mg; Folic acid 16 mg; Biotin 1.6 mg; Iron 2–10 g; Copper 0.1–0.6 g; Zinc 0.5–2.0 g; Manganese 0.5–2.5 g; Iodine 5–50 mg; Selenium 3–12 mg; Calcium 5–30%; Total phosphorus 1.4%; Sodium chloride 8–24%; Choline chloride 5%; Lysine 1.8%; Moisture 10%.

**Table 2:** Impact of high-fiber diet on the growth performance of JW.

Item	CON	EG	*p*-Value	SEM
Initial body weight (kg)	49.51 ± 7.51	51.67 ± 7.10	0.221	1.20
Final body weight (kg)	96.49 ± 10.30	95.53 ± 10.16	0.697	1.72
Overall average daily gain (kg/d)	0.72 ± 0.14	0.67 ± 0.11	0.109	0.02
Overall average daily feed intake (kg/d)	26.54 ± 13.68	28.17 ± 12.42	0.865	6.21
Overall feed conversion Ratio (F/G)	3.52 ± 0.12	3.67 ± 0.16	0.734	0.89

Abbreviations: CON, basal diet group; EG, roughage diet group; *p*-value, values with different letters differ significantly (*p* < 0.05); SEM, standard error of the mean.

**Table 3:** Impact of high-fiber diet on the growth performance of DLY.

Item	CON	EG	*p*-Value	SEM
Initial body weight (kg)	57.00 ± 8.82	57.11 ± 11.06	0.964	1.84
Final body weight (kg)	116.59 ± 17.17	112.53 ± 18.82	0.354	3.14
Overall average daily gain (kg/d)	0.92 ± 0.18	0.85 ± 0.16	0.130	0.03
Overall average daily feed intake (kg/d)	26.35 ± 10.15	29.71 ± 13.20	0.700	6.59
Overall feed conversion ratio (F/G)	3.01 ± 0.27	3.54 ± 0.31	0.076	1.24

Abbreviations: CON, basal diet group; EG, roughage diet group; *p*-value, values with different letters differ significantly (*p* < 0.05); SEM, standard error of the mean.

**Table 4:** Two-factor analysis of variance table for daily weight gain traits of JW and DLY.

Source	DF	Sum of Squares	Mean Square	F Value	*Pr* > F
Model	3	1.2845	0.4285	18.78	<0.0001
Pig	1	1.1706	1.1706	51.35	<0.0001
Feed	1	0.1008	0.1008	4.42	0.0374
Pig × feed	1	0.0131	0.1311	0.58	0.4495
Error	134	3.0547	0.0228		

## Data Availability

The data that support the findings in this study were not deposited in an official repository. These data are available from the authors upon request.
